# Disrupted Regional Homogeneity in Melancholic and Non-melancholic Major Depressive Disorder at Rest

**DOI:** 10.3389/fpsyt.2021.618805

**Published:** 2021-02-16

**Authors:** Meiqi Yan, Yuqiong He, Xilong Cui, Feng Liu, Huabing Li, Renzhi Huang, Yanqing Tang, Jindong Chen, Jingping Zhao, Guangrong Xie, Wenbin Guo

**Affiliations:** ^1^Department of Psychiatry, National Clinical Research Center for Mental Disorders, The Second Xiangya Hospital of Central South University, Changsha, China; ^2^Department of Radiology, Tianjin Medical University General Hospital, Tianjin, China; ^3^Department of Radiology, The Second Xiangya Hospital of Central South University, Changsha, China; ^4^Hunan Key Laboratory of Children's Psychological Development and Brain Cognitive Science, Changsha, China; ^5^Department of Psychiatry, The First Affiliated Hospital of China Medical University, Shenyang, China; ^6^Department of Psychiatry, The Third People's Hospital of Foshan, Foshan, China

**Keywords:** melancholic depression, non-melancholic depression, regional homogeneity, magnetic resonance imaging, resting state

## Abstract

**Background:** Melancholic depression has been viewed as one severe subtype of major depressive disorder (MDD). However, it is unclear whether melancholic depression has distinct changes in brain imaging. We aimed to explore specific or distinctive alterations in melancholic MDD and whether the alterations could be used to separate melancholic MDD from non-melancholic MDD or healthy controls.

**Materials and Methods:** Thirty-one outpatients with melancholic MDD and thirty-three outpatients with non-melancholic MDD and thirty-two age- and gender-matched healthy controls were recruited. All participants were scanned by resting-state functional magnetic resonance imaging (fMRI). Imaging data were analyzed with the regional homogeneity (ReHo) and support vector machine (SVM) methods.

**Results:** Melancholic MDD patients exhibited lower ReHo in the right superior occipital gyrus/middle occipital gyrus than non-melancholic MDD patients and healthy controls. Merely for non-melancholic MDD patients, decreased ReHo in the right middle frontal gyrus was negatively correlated with the total HRSD-17 scores. SVM analysis results showed that a combination of abnormal ReHo in the right fusiform gyrus/cerebellum Crus I and the right superior occipital gyrus/middle occipital gyrus exhibited the highest accuracy of 83.05% (49/59), with a sensitivity of 90.32% (28/31), and a specificity of 75.00% (21/28) for discriminating patients with melancholic MDD from patients with non-melancholic MDD. And a combination of abnormal ReHo in the right fusiform gyrus/cerebellum VI and left postcentral gyrus/precentral gyrus exhibited the highest accuracy of 98.41% (62/63), with a sensitivity of 96.77% (30/31), and a specificity of 100.00%(32/32) for separating patients with melancholic MDD from healthy controls.

**Conclusion:** Our findings showed the distinctive ReHo pattern in patients with melancholic MDD and found brain area that may be associated with the pathophysiology of non-melancholic MDD. Potential imaging markers for discriminating melancholic MDD from non-melancholic MDD or healthy controls were reported.

## Introduction

Major depressive disorder (MDD), a common, recurrent and disabling psychiatric disorder, severely leads to impaired psychosocial function and reduced quality of life (1). Melancholic depression has been viewed as one severe subtype of MDD. MDD is characterized by a persistent depressed mood, anxiety and dysphoria, psychomotor changes, alterations of motivation and social behavior, and sleep abnormalities (2). Despite of the common features of MDD, melancholic depression is characterized by pervasive anhedonia, unremitting apprehension and morbid statements, blunted emotional response, non-reactive mood, retardation, spontaneous agitation, reduced concentration, and impaired working memory (3). Although there is no consensus on whether melancholic depression is a categorically separate psychiatric disorder or a dimensionally severe subtype of MDD (4, 5), poorer cognitive performance was observed in melancholic MDD than non-melancholic MDD, particularly in visual learning and executive functions, like attention/working memory, reasoning/problem solving and processing speed (6, 7). The periods of cognitive recovery need longer time in melancholic MDD patients than the non-melancholic individuals (8). Besides, patients with melancholic MDD showed higher risk of relapse than patients with non-melancholic MDD (9). Thus, the diagnosis of melancholic MDD has a superior predictive validity for its treatment and prognosis (3).

Patients with MDD have been reported to show structural and functional abnormalities in brain imaging, like abnormal network homogeneity (NH) (10–12), functional connectivity (FC) (13–15), voxel-mirrored homotopic connectivity (VMHC) (16–18), low-frequency fluctuations (ALFF) (19, 20), voxel-based morphometry (VBM) (21, 22) and functional network connectivity (FNC) (23, 24). Meanwhile, structural or functional alterations in melancholic patients have been revealed in many previous studies, such as gray/white matter alterations (25), asymmetrical enlargement of CSF space in the Sylvian fissure region (26), decreased effective connectivity of cortical systems involved in attention and interoception compared with non-melancholic MDD patients and healthy controls (27), lower mean fractional anisotropy (FA) in the right ventral tegmental area-lateral orbitofrontal cortex (VTA-LOFC) connection than patients with non-melancholic MDD (28). Increased FA and decreased radial diffusivity (RD) were also identified in the right anterior limb of the internal capsule in melancholic MDD compared with health controls (29). A previous functional connectivity study reported that melancholic medication-free remitted MDD (rMDD) patients exhibited decreased subgenual cingulate cortex (SCC) connectivity with the parahippocampal gyrus and amygdala compared with non-melancholic rMDD patients (30). However, it is unclear which brain region is abnormal when one brain region shows abnormal functional connections to other brain regions.

Regional homogeneity (ReHo) method reflects the temporal homogeneity of the regional blood oxygen level-dependent (BOLD) signal that enables it to reveal the temporal homogeneity of neural activity. A previous Reho study believed that voxels within a functional brain region were more temporally homogeneous involving in a certain condition (31). So abnormal ReHo may be due to unsynchronized regional neural activity, and the ReHo method can be applied to find abnormal neural activity in the whole brain regions. Kendall's coefficient of concordance (KCC) can measure the similarity of multiple time series. KCC was applied to measure ReHo of the time series of one given voxel with those of its nearest voxels in a voxel-wise way (32, 33). Many previous studies have applied the ReHo method to study psychiatric disorders such as depression (34, 35), schizophrenia (36) and ADHD (37). However, the questions of whether melancholic MDD has specific or distinctive alterations relative to non-melancholic MDD in regional homogeneity and whether abnormal regional homogeneity could be used to distinguish melancholic MDD and non-melancholic MDD as well as healthy controls still remain unknown.

To address these questions, we applied the ReHo method to examine patients with melancholic MDD and patients with non-melancholic MDD. We hypothesized that abnormal ReHo would be observed in certain regions in patients with melancholic MDD at rest and abnormal regions might be distinct areas that could be used to discriminating melancholic MDD from non-melancholic MDD or healthy controls. To test our hypotheses, we compared ReHo of whole brain regions across patients with melancholic MDD, patients with non-melancholic MDD and healthy controls.

## Methods

### Participants

We recruited a total of 31 outpatients with melancholic MDD and 33 outpatients with non-melancholic MDD of the Second Xiangya Hospital of Central South University. They were all aged from 18 to 45 years old. The data were collected from May 4, 2014 to December 30, 2016. The diagnosis was independently ascertained by two psychiatrists, according to the Diagnostic and Statistical Manual of Mental Disorders, Fourth Edition (DSM-4). All the patients met the following inclusion criteria: (1) first major depressive episode with Hamilton Rating Scale for Depression (HRSD-17) (38) total scores ≥17 and HRSD item 1 score ≥ 2; (2) illness duration <12 months; (3) no history of antipsychotics and electroconvulsive therapy. The criteria for the features of melancholic MDD in DSM-4 were required as follows: (1) at least one of the following symptoms occurs during the most severe period of the current episode: loss of pleasure in all or almost all activities (pervasive anhedonia); lack of mood reactivity to usually pleasurable stimuli (does not feel much better, even temporally, when something good happens) (non-reactive mood); (2) three (or more) of the following symptoms: distinct quality of depressed mood (i.e., depressive mood experienced is qualitatively different from the feeling experienced when the loved one dies. Showing extreme despondency, despair, and/or morose mood or alleged empty mood); depression is regularly worse in the morning; early morning awakening (i.e., at least 2 h earlier than usual awakening) (HRSD item 6 ≥ 1); marked psychomotor agitation or retardation (HRSD items 8 or 9 ≥ 2); significant anorexia or weight loss (HRSD items 12 or 16 = 2); and excessive or inappropriate guilt (HRSD item 2 ≥ 2). The anhedonic states was assessed by using the Chinese version of Snaith-Hamilton Pleasure Scale (SHAPS-C) (39), the higher the score, the more severe the anhedonia; The Chinese version of Temporal Experience of Pleasure Scale (TEPS) was applied to capture the level of anticipatory and consummatory facets of pleasure, the lower the score, the greater the anhedonia. Non-melancholic MDD group consisted of patients who did not meet these criteria.

A total of 32 age- and gender-matched healthy controls were recruited from the community. Healthy controls would be excluded if they were related to the patients or had a family history of mental illness, especially their first-degree relatives. Besides, they would be ruled out if they had any neurological disorders, substance abuse or psychosis symptoms.

Exclusion criteria for all participants as follows: (1) other psychiatric disorders meeting DSM-4 diagnostic criteria; (2) any history of neurological disorders, severe physical illnesses, and substance abuse; (3) pregnancy; (4) abnormal cerebral structure after initial MRI scan; (5) any contraindications for MRI scan.

All participants were right-handed and Han Chinese with at least 9 years of education. The HRSD-17 was applied to determine the severity of depression; The Beck anxiety inventory (BAI) was administered to evaluate anxiety state. All participants were evaluated by the HRSD-17, SHAPS-C and BAI, and all patients completed TEPS evaluation.

The study was approved by the Medical Research Ethics Committee of the Second Xiangya Hospital of Central South University, China. The study was performed in line with the Helsinki Declaration. Each participant has completed an informed consent prior to enrollment.

### Image Acquisition

Resting-state MRI data were obtained with a 3.0 T Siemens scanner (Germany) at the Second Xiangya Hospital of Central South University. All subjects were asked to lay supine, remain still, close their eyes and stay awake. Soft earplugs and foam pads were used to reduce scanner noise and head movement. Using the echo planar imaging (EPI) sequence, the resting-state functional images were obtained through the following parameters: repetition time/echo time (TR/TE) 2,500/25 ms, 39 slices, 64^*^64 matrix, 90°flip angle, 24 cm field of view, 3.5 mm slice thickness, no gap, and 200 volumes lasting for 500 s.

### Data Preprocessing

Data preprocessing was conducted in Matlab (Mathworks) by using Data Processing Assistant for Resting-State fMRI (DPARSF) (40). Considering the instability of the initial MRI signal and the influences of subjects' adaptation time, the first 10 images were discarded. None of the participants had more than 2 mm of maximum displacement in x, y, or z axis or more than 2 of angular rotation after correction for slice timing and head motion. Then, the corrected imaging data were spatially normalized to the MNI space and got resampled to 3 × 3 × 3 mm^3^. After that, the fMRI data were temporally band-pass filtered (0.01–0.08 Hz) and linearly detrended. Several spurious covariates were also removed, such as the signal from the white matter-centered region and the ventricular seed-based region of interest (ROI) (Supplementary Figure 1) as well as the 24-head motion parameters obtained by rigid body correction. According to a previous study (41), global signal was retained when the resting-state functional connectivity (FC) data was pre-processed.

### ReHo Analysis

Regional homogeneity (ReHo) analysis was conducted by in-house software REST (http://www.resting-fmri.sourceforge.net). Individual ReHo maps were generated by calculating the KCC of the time series of a given voxel with those of its nearest voxels (26 voxels) in a voxel-wise analysis. The formula for calculating the KCC value has been elaborated in a previous study (31). In order to reduce the influence of individual variation in the KCC value, we normalized the ReHo maps by dividing the KCC of each voxel by the averaged KCC of the whole brain. The resulting imaging data were then spatially smoothed with a Gaussian kernel of 4 mm full-width at half-maximum.

### Statistical Analyses

Analysis of variance (ANOVA) was conducted to analyze group differences in age, years of education, HRSD-17 scores, BAI scores and SHAPS-C scores across the three groups by using SPSS19.0 (LSD between two group comparison), and we applied two-sample *t-*test to analyze whether there were group differences in the illness duration and the TEPS scores between melancholic MDD group and non-melancholic MDD group. A Chi-square test was performed to describe gender distributions. The significance level was set at *p* < 0.05.

Analysis of covariance (ANCOVA) was performed on individual whole brain ReHo maps across the three groups in a voxel-by-voxel manner to identify the group differences, followed by *post-hoc t-*tests. Age, years of education and framewise displacement were used as covariates. The results were FDR (false discovery rate) corrected at *p* < 0.05.

### Correlation Analyses

The voxel-based mean ReHo values were extracted from the brain regions with abnormal ReHo by in-house software REST (http://www.resting-fmri.sourceforge.net). The correlations between abnormal ReHo and HRSD-17, BAI, SHARS, TEPS scores were determined using Pearson's correlation analyses with a threshold of Benjamini-Hochberg corrected *p* < 0.05.

### Classification Analyses

In the LIBSVM software package (http://www.csie.ntu.edu.tw/~cjlin/libsvm/) in MATLAB, support vector machines (SVM) was applied to test the ability to separate melancholic MDD from non-melancholic MDD, melancholic MDD from healthy controls, and non-melancholic MDD from healthy controls by using the identified ReHo values in abnormal brain regions. The “leave-one-out” method was applied in the study.

## Results

### Demographic Characteristics and Clinical Information

Five patients with non-melancholic MDD were excluded due to excessive head movement. Thus, 31 patients with melancholic MDD, 28 patients with non-melancholic MDD and 32 healthy controls were finally included in the analyses. Age and gender did not significantly differ across the three groups, and no illness duration difference was observed between the two patient groups. Significant differences were observed across the three groups in years of education, HRSD-17 scores, BAI scores and SHAPS-C scores. The melancholic MDD group (*p* = 0.001) and the healthy control group (*p* = 0.01) showed significantly higher education level than that of the non-melancholic MDD group. The HRSD-17, BAI and SHAPS-C scores of the melancholic MDD group (*p* < 0.001) group and non-melancholic MDD group (*p* < 0.001) were significantly higher than those of the healthy control group. The non-melancholic group exhibited significantly lower BAI (*p* = 0.024) and SHAPS-C scores (*p* < 0.001) than those of the melancholic group, while no significant difference in HRSD-17 scores was observed between the two patient groups (*p* = 0.743). Part of the TEPS scores showed significant differences between the two patient groups. The TEPS total scores (*p* = 0.002), TEPS Abstract Anticipatory scores (*p* = 0.001) and TEPS Contextual Anticipatory scores (*p* = 0.001) of the melancholic group were significantly lower than those of the non-melancholic group. The TEPS Abstract Consummatory scores (*p* = 0.117) and TEPS Contextual Consummatory (*p* = 0.074) did not significantly differ between the two patient groups. More details of demographic and clinical data are presented in Table 1.

**Table 1:** Demographic and clinical characteristics of the participants.

	**Melancholic** ** (*n =* 31)**	**Non-melancholic** ** (*n =* 28)**	**Healthy controls** ** (*n =* 32)**	***F,t*** ** or *χ^2^* Value**	***P*-Value** ** (two-tailed)**
Age (years)	28.65 ± 5.30	32.04 ± 8.18	29.59 ± 5.00	2.291	0.107^a^
Gender (male/female)	10/21	10/18	15/17	1.55	0.461^b^
Handedness (Right/Left)	31/0	28/0	32/0		
Education (years)	15.16 ± 3.20	12.54 ± 3.00	14.59 ± 2.82	6.143	0.003^a^
Illness duration (months)	6.75 ± 4.26	5.96 ± 4.64		−0.68	0.500^c^
HRSD-17 scores	21.77 ± 3.79	21.00 ± 3.14	0.94 ± 0.95	527.891	<0.001^a^
BAI scores	44.00 ± 11.51	38.77 ± 9.84	22.63 ± 2.28	50.895	<0.001^a^
SHAPS-C scores	37.23 ± 6.04	31.89 ± 5.24	21.59 ± 5.36	64.191	<0.001^a^
TEPS total scores	58.30 ± 14.19	69.46 ± 11.16		−3.315	0.002^c^
TEPS Abstract Anticipatory	13.17 ± 4.79	17.04 ± 3.85		−3.373	0.001^c^
TEPS Contextual Anticipatory	13.13 ± 3.96	16.68 ± 3.64		−3.540	0.001^c^
TEPS Abstract Consummatory	20.20 ± 5.21	22.39 ± 5.28		−1.592	0.117^c^
TEPS Contextual Consummatory	11.80 ± 3.23	13.36 ± 3.27		−1.824	0.074^c^

*HRSD-17, 17-item Hamilton Rating Scale for Depression; BAI, Beck anxiety inventory; SHAPS-C, Chinese version of Snaith–Hamilton Pleasure Scale*.

a *the p-value was obtained by analyses of variance*.

b *the p-value was obtained by a chi-square test*.

c *the p-value was obtained by two-sample t-tests*.

### ReHo Differences Between Groups

**Figure 1:**
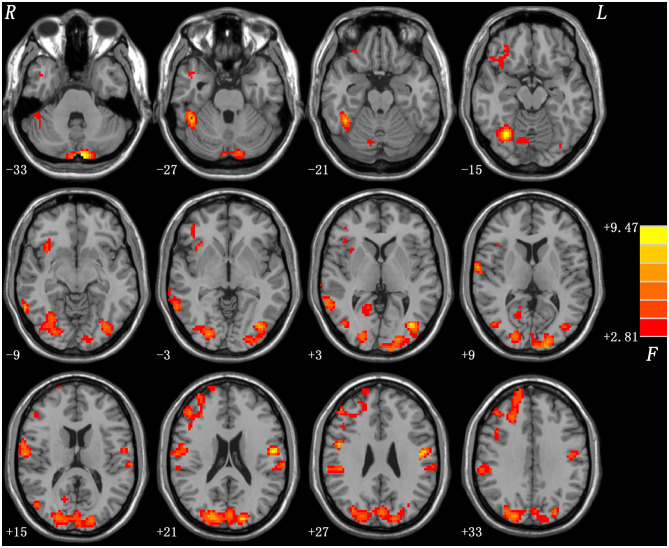
Brain regions showing group differences of ReHo values across three groups. Color bar indicates F values from ANCOVA (age, years of education and framewise displacement as covariates). ReHo, regional homogeneity; ANCOVA, analysis of covariance.

By using ANCOVA, the brain regions mainly in the right middle frontal gyrus, right fusiform gyrus/cerebellum Crus I, bilateral cerebellum Crus II, right fusiform gyrus/cerebellum VI, left middle occipital gyrus/inferior occipital gyrus, bilateral superior occipital gyrus/middle occipital gyrus, bilateral postcentral gyrus/precentral gyrus and right middle temporal gyrus showed significant differences of ReHo values across the three groups (Figure 1). We reanalyzed ANCONA adding gender as a covariate since previous studies (42–44) have reported the gender difference in depression, which is more prone to females. Similar results were obtained (Supplementary Table 1).

#### Melancholic vs. Non-melancholic

**Figure 2:**
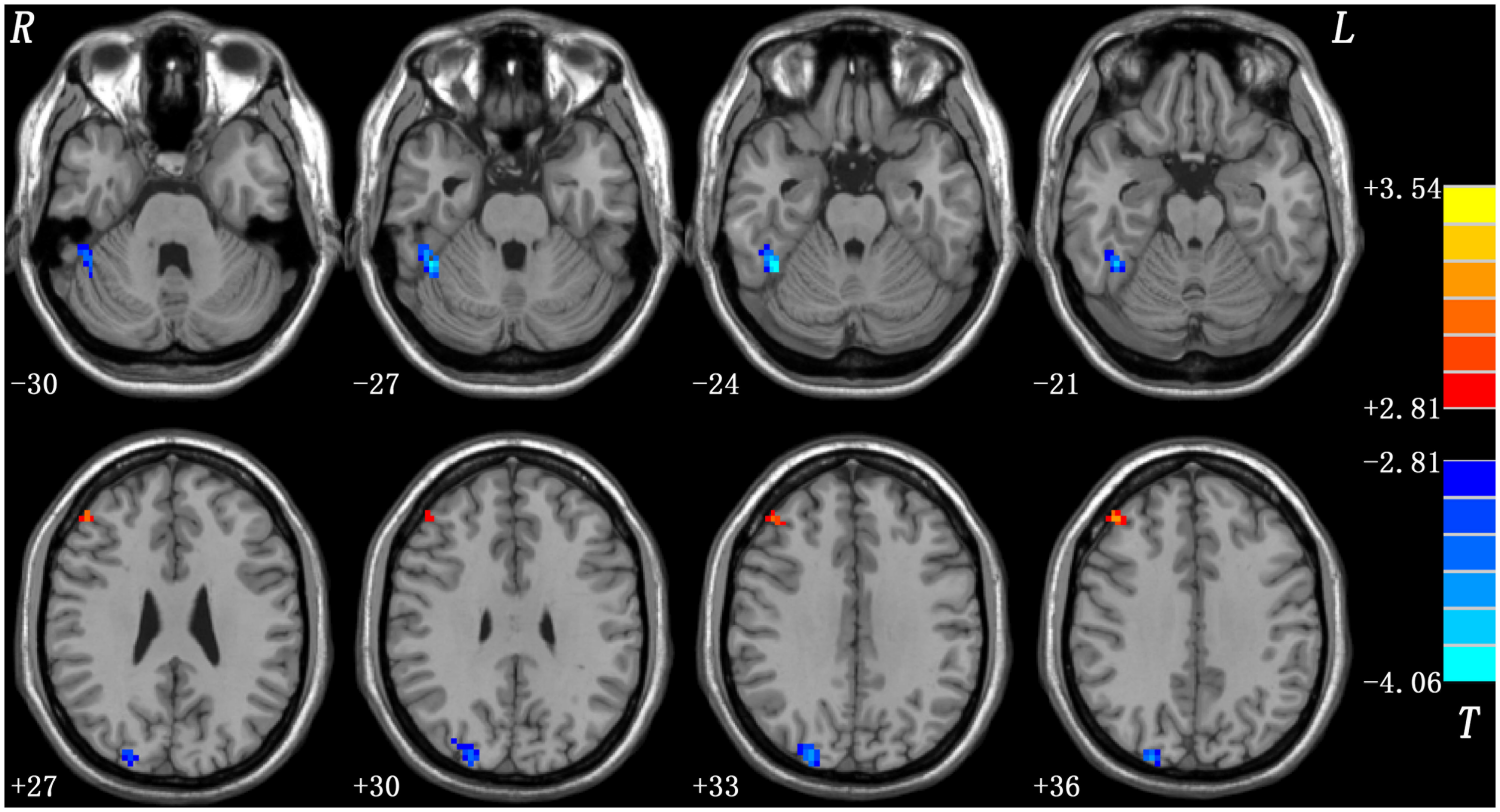
Statistical map depicts higher and lower ReHo of melancholic MDD patients compared with non-melancholic MDD patients. The threshold was set at *p* < 0.05. Blue denotes lower ReHo and red denotes higher ReHo. Color bar indicates T values from *post-hoc t-*tests. L, left side; R, right side; ReHo, regional homogeneity.

Compared with non-melancholic group, melancholic group showed increased ReHo in the right middle frontal gyrus and decreased ReHo in the right fusiform gyrus/cerebellum Crus I and the right superior occipital gyrus/middle occipital gyrus (Figure 2, Table 2).

**Table 2:** Significant ReHo differences across three groups.

**Cluster location**	**Peak (MNI)**			**Number of voxels**	***T*-value**
	**x**	**y**	**z**		
***Melancholic vs. Non-melancholic***					
Right Middle Frontal Gyrus	42	39	36	38	3.2503
Right Fusiform Gyrus/Cerebellum Crus I	42	−48	−24	51	−4.0602
Right Superior Occipital Gyrus/Middle Occipital Gyrus	24	−90	33	71	−3.5526
***Melancholic vs. Healthy Controls***					
Bilateral Cerebellum Crus II	−9	−90	−33	51	4.1721
Right Fusiform Gyrus/Cerebellum VI	30	−63	−15	62	−4.4930
Left Middle Occipital Gyrus/Inferior Occipital Gyrus	−36	−75	3	87	−4.1830
Bilateral Superior Occipital Gyrus/Middle Occipital Gyrus	18	−87	21	325	−3.6261
Right Postcentral Gyrus/Precentral Gyrus	63	−9	15	31	−3.6665
Left Postcentral Gyrus/Precentral Gyrus	−54	−9	21	64	−4.6822
***Non-melancholic vs. Healthy Controls***					
Right Middle Temporal Gyrus	63	−51	0	38	3.4241

*MNI, Montreal Neurological Institute*.

*ReHo, regional homogeneity*.

#### Melancholic vs. Healthy Controls

**Figure 3:**
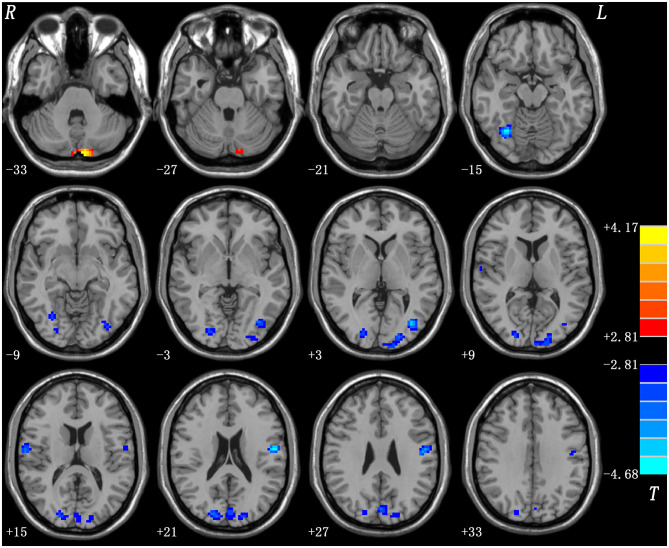
Statistical map depicts higher and lower ReHo of melancholic MDD patients compared with healthy controls. The threshold was set at *p* < 0.05. Blue denotes lower ReHo and red denotes higher ReHo. Color bar indicates T values from *post-hoc t-*tests. L, left side; R, right side; ReHo, regional homogeneity.

Compared with healthy control group, melancholic group exhibited increased ReHo in the bilateral cerebellum Crus II and decreased ReHo in the right fusiform gyrus/cerebellum VI, left middle occipital gyrus/inferior occipital gyrus, bilateral superior occipital gyrus/middle occipital gyrus, and bilateral postcentral gyrus/precentral gyrus (Figure 3, Table 2).

#### Non-melancholic vs. Healthy Controls

**Figure 4:**
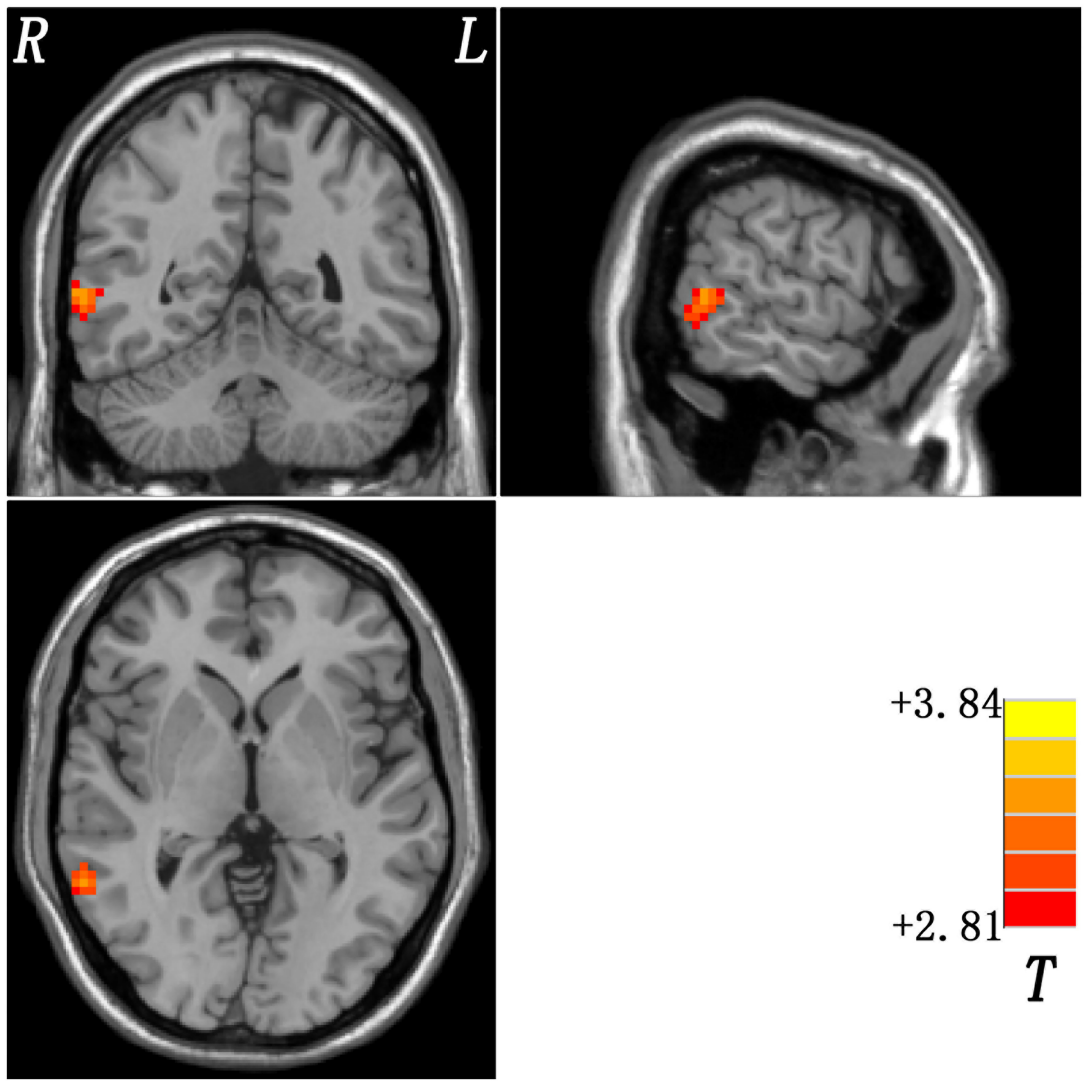
Statistical map depicts higher ReHo of non-melancholic MDD patients compared with healthy controls. The threshold was set at *p* < 0.05. Red denotes higher ReHo. Color bar indicates T values from *post-hoc t-*tests. L, left side; R, right side; ReHo, regional homogeneity.

Compared with healthy control group, increased ReHo in the right middle temporal gyrus was found in non-melancholic group (Figure 4, Table 2).

### Correlations Between ReHo and Clinical Characteristics

No significant correlation between abnormal ReHo and clinical features was found in melancholic MDD patients. For non-melancholic MDD patients, decreased ReHo in the right middle frontal gyrus was negatively correlated with the total HRSD-17 scores (*r* = −0.527, *p* = 0.004, Benjamini-Hochberg correction *p* = 0.016).

### Discrimination Patients With Melancholic MDD From Other Two Groups

#### Discriminating Patients With Melancholic MDD From Non-melancholic MDD

**Figure 5:**
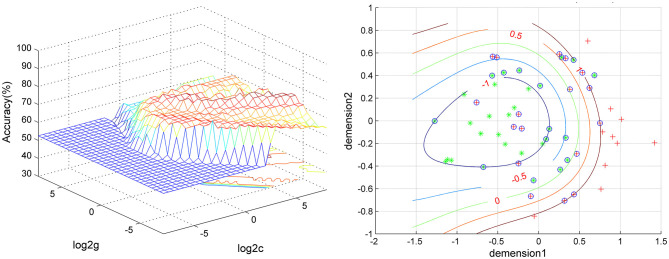
Visualization of classifications through support vector machine (SVM) using the combination of ReHo values in the right fusiform gyrus/cerebellum Crus I and the right superior occipital gyrus/middle occipital gyrus to discriminate melancholic MDD and non-melancholic MDD. Left: SVM parameters result of 3D view. Right: dimension 1 and dimension 2 represent the ReHo values in the right fusiform gyrus/cerebellum Crus I and the right superior occipital gyrus/middle occipital gyrus, respectively. Red crosses represent non-melancholic MDD patients, and green crosses represent the melancholic MDD patients. MDD, major depression disorder.

**Figure 6:**
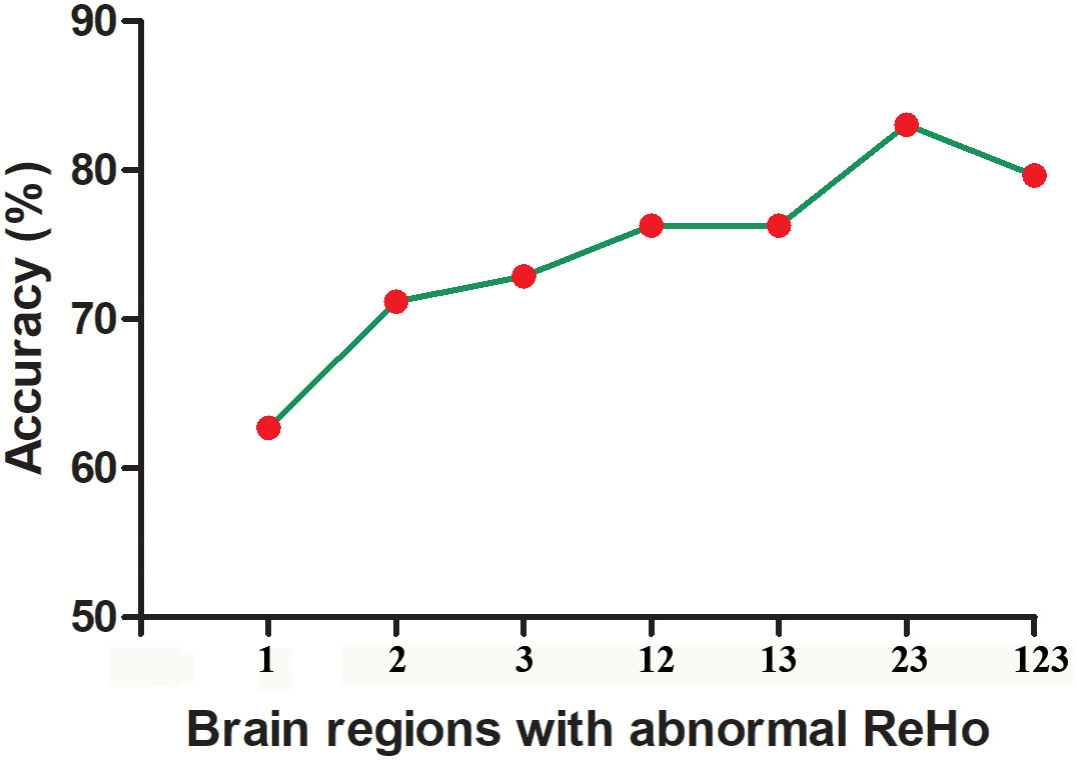
The accuracy of using abnormal ReHo in different brain regions to classify patients with melancholic and non-melancholic MDD. 1, Right Middle Frontal Gyrus; 2, Right Fusiform Gyrus/Cerebellum Crus I; 3, Right Superior Occipital Gyrus/Middle Occipital Gyrus; 12, Right Middle Frontal Gyrus and Right Fusiform Gyrus/Cerebellum Crus I; 13, Right Middle Frontal Gyrus and Right Superior Occipital Gyrus/Middle Occipital Gyrus; 23, Right Fusiform Gyrus/Cerebellum Crus I and Right Superior Occipital Gyrus/Middle Occipital Gyrus; 123, Right Middle Frontal Gyrus and Right Fusiform Gyrus/Cerebellum Crus I and Right Superior Occipital Gyrus/Middle Occipital Gyrus.

The SVM results showed that a combination of abnormal ReHo in the right fusiform gyrus/cerebellum Crus I and right superior occipital gyrus/middle occipital gyrus exhibited the highest accuracy of 83.05% (49/59), with a sensitivity of 90.32% (28/31), and a specificity of 75.00%(21/28) for discriminating patients with melancholic MDD from patients with non-melancholic MDD (Figures 5, 6).

#### Discriminating Patients With Melancholic MDD From Healthy Controls

**Figure 7:**
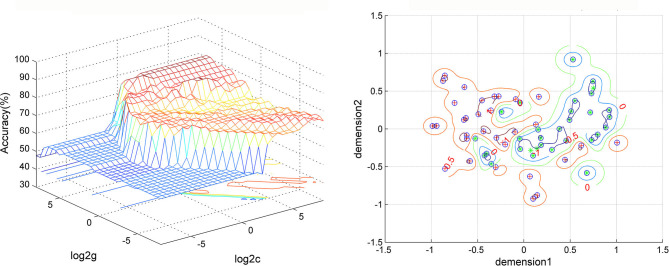
Visualization of classifications through support vector machine (SVM) using the combination of ReHo values in the right fusiform gyrus/cerebellum VI and left postcentral gyrus/precentral gyrus to discriminate melancholic MDD and from controls. Left: SVM parameters result of 3D view. Right: dimension 1 and dimension 2 represent the ReHo values in the right fusiform gyrus/cerebellum VI and left postcentral gyrus/precentral gyrus, respectively. Red crosses represent healthy controls, and green crosses represent the melancholic MDD patients. MDD, major depression disorder.

The SVM results showed that a combination of abnormal ReHo in the right fusiform gyrus/cerebellum VI and left postcentral gyrus/precentral gyrus showed the highest accuracy of 98.41% (62/63), with a sensitivity of 96.77% (30/31), and a specificity of 100.00% (32/32) for distinguishing patients with melancholic MDD from healthy controls (Figure 7).

### Discriminating Patients With Non-melancholic MDD From Healthy Controls

**Figure 8:**
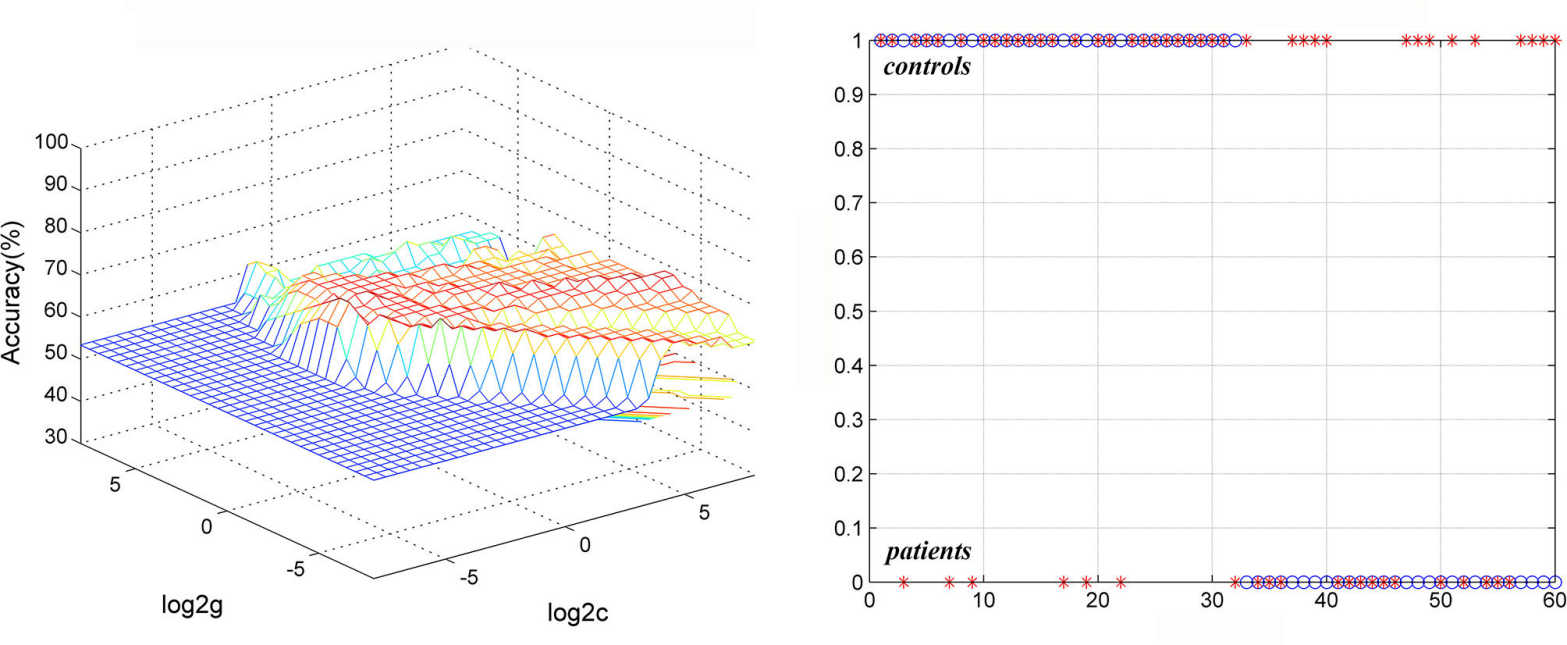
Visualization of classifications through support vector machine (SVM) using the increased ReHo values in the right middle temporal gyrus to discriminate non-melancholic MDD patients from healthy controls. Left: SVM parameters result of 3D view. Right: Classified map of the ReHo values in the right middle temporal gyrus. MDD, major depressive disorder.

The SVM results showed that abnormal ReHo in the right middle temporal gyrus had an accuracy of 65% (39/60), with a sensitivity of 50% (14/28), and a specificity of 78.13% (28/32) for separating the non-melancholic MDD patients from healthy controls (Figure 8).

## Discussion

In the present study, we investigated the whole brain regional homogeneity in patients with melancholic MDD and non-melancholic MDD at rest. We observed that melancholic MDD patients exhibited decreased ReHo in the right superior occipital gyrus/middle occipital gyrus compared with non-melancholic MDD patients and healthy controls. Abnormal ReHo in the right middle temporal gyrus may play an important role in the pathophysiology of non-melancholic MDD. Merely for non-melancholic MDD patients, decreased ReHo in the right middle frontal gyrus was negatively correlated with the total HRSD-17 scores. In addition, the SVM analysis results showed that a combination of abnormal ReHo in the right fusiform gyrus/cerebellum Crus I and right superior occipital gyrus/middle occipital gyrus might be a potential imaging marker to separate melancholic MDD patients from non-melancholic MDD patients, and a combination of abnormal ReHo in the right fusiform gyrus/cerebellum VI and left postcentral gyrus/precentral gyrus might be a potential imaging marker to separate melancholic MDD patients from healthy controls.

Occipital cortex involves in consolidating information into visual working memory (45). A previous study has observed that the occipital bending in MDD patients was more common than healthy controls and enlargement of ventricle may exacerbate the natural curvature of the occipital regions (46). Another study has revealed the asymmetrical enlargement of CSF space in the Sylvian fissure region in melancholic MDD patients (26). That issue might indicate that abnormal structural or functional changes may occur in the occipital regions in melancholic MDD. Consistent with these studies, we found that melancholic MDD patients exhibited decreased ReHo in the right superior occipital gyrus/middle occipital gyrus compared with non-melancholic MDD patients and healthy controls. And that point can interpret working memory impairment in melancholic MDD (45). Hence, we can speculate that decreased ReHo in the right superior occipital gyrus/middle occipital gyrus might be stable and distinctive neurobiological characteristic of melancholic MDD.

The temporal lobe has been expounded to get involved in emotional regulation, process of memory, and social cognition (47, 48). Many previous studies have observed abnormal brain activity or functional connectivity in the temporal areas in MDD patients. Decreased NH was found in melancholic MDD in the right middle temporal gyrus and temporal pole (MTG/TP) compared with healthy controls (12). MDD patients showed lower NH in the right inferior temporal gyrus (ITG) than healthy controls (10). Remitted geriatric depression exhibited decreased ReHo in the right superior and middle temporal gyrus (49). MDD showed significantly increased ReHo in the right inferior temporal gyrus (50). In the present study, we found increased ReHo of the right middle temporal gyrus in non-melancholic MDD patients compared with healthy controls. Although we could not directly compare these above mentioned studies because of different composition of MDD patients, these studies indicated abnormal brain activity of temporal gyrus in non-melancholic MDD. We did not find abnormal ReHo in the temporal gyrus in patients with melancholic MDD compared with both healthy controls and non-melancholic MDD patients. We suspected that the differences may be that the non-melancholic patients were characterized by other main characteristics (like anxiety, atypical increased sleep, somatic symptoms, etc.) rather than melancholic traits. A voxel-based morphometry (VBM) study reported that anxiety was positive correlated with VBM abnormalities in the middle temporal gyrus (51). A ReHo study of MDD patients with somatic symptoms (somatic depression, SD) reported that abnormal ReHo in the frontal and temporal regions may be involved in the neural basis of SD (52). Some previous studies revealed that the structural alterations in the temporal gyrus might be associated with suicide attempt (53, 54). Although we did not further classify the subtypes of non-melancholic patients because of small sample size, the present study reminded us to examine this issue in the future study. Thus, we speculated that abnormal ReHo in the right middle temporal gyrus may play an important role in the pathophysiology of non-melancholic MDD.

Frontal lobe is associated with cognitive control (55). Abnormal structural and functional alterations in the frontal gyrus in patients MDD were reported in numerous previous studies (56–58). Melancholic MDD was reported to show cognitive impairment (like poorer processing speed and verbal fluency) than HCs and atypical depression (59). A distinct and poorer cognitive performance was also reported in melancholic MDD compared with non-melancholic MDD (6, 7). In present study, non-melancholic group showed decreased ReHo in the right middle frontal gyrus compared with melancholic group and decreased ReHo in the right middle frontal gyrus was negatively correlated with the total HRSD-17 scores in non-melancholic MDD patients. The significant differences in the frontal gyrus between two patient groups might interpret poorer cognitive performance in melancholic MDD compared with non-melancholic MDD (6).

Support vector machine (SVM) has been widely used in biomedical applications for diagnoses of psychiatric disorders like major depression (60, 61), schizophrenia (62) and others (63). The sensitivity or specificity of qualified diagnostic indicators should not be <60%, and more than 70% is conducive to the establishment of diagnostic indicators (64, 65). The SVM analysis results showed that a combination of abnormal ReHo in the right fusiform gyrus/cerebellum Crus I and the right superior occipital gyrus/middle occipital gyrus exhibited the highest accuracy of 83.05% (49/59), with a sensitivity of 90.32% (28/31), and a specificity of 75.00% (21/28) for discriminating patients with melancholic MDD from patients with non-melancholic MDD. Thus, the combination of ReHo in the right fusiform gyrus/cerebellum Crus I and the right superior occipital gyrus/middle occipital gyrus might be employed as a potential imaging marker for discriminating patients with melancholic MDD from patients with non-melancholic MDD. The melancholic MDD is characterized by pervasive anhedonia and non-reactive mood, etc. (as we mentioned in the introduction part) (3), and shows poorer cognitive performance than non-melancholic MDD (6, 7). In the present study, non-melancholic patients were composited of other subtype of MDD instead of melancholic subtype, and were characterized by other characteristics (such as anxiety, atypical increased sleep, somatic symptoms, etc.) rather than melancholic traits. This may be the main reason for the apparent difference between the two subtypes of MDD. Besides, to increase the accuracy, we could apply multimodal brain imaging data to discriminate the two subtypes of MDD in future studies according to a previous study (66), such as combining two types of functional data, or combining both anatomical and functional data. In the present study, the SVM results showed that a combination of abnormal ReHo in the right fusiform gyrus/cerebellum VI and left postcentral gyrus/precentral gyrus exhibited the highest accuracy of 98.41% (62/63), with a sensitivity of 96.77% (30/31), and a specificity of 100.00% (32/32) for separating patients with melancholic MDD from healthy controls. Thus, we speculated that the combination of abnormal ReHo in the right fusiform gyrus/cerebellum VI and left postcentral gyrus/precentral gyrus might be a potential imaging marker for separating melancholic MDD patients from healthy controls. When we used abnormal ReHo in the right middle temporal gyrus to separate non-melancholic MDD from healthy controls, we obtained a moderate accuracy of 65% (39/60), with a sensitivity of 50% (14/28) and a specificity of 78.13% (28/32), which might be weak for the classification due to the lower sensitivity. As we mentioned earlier, the different composition of non-melancholic patients may have an impact on this issue, and we can further classify these patients into different subtypes to explore them in future studies.

Some limitations exist in our study. First, our sample size is small. Second, we recruited both melancholic and non-melancholic MDD patients but we did not further classify the non-melancholic MDD patients due to small sample size. Therefore, we were unable to further explore the neurobiological differences between different subtypes of non-melancholic MDD.

## Conclusion

Our findings showed the distinctive ReHo pattern in patients with melancholic MDD. Decreased ReHo in the right superior occipital gyrus/middle occipital gyrus might be a stable and distinctive neurobiological characteristic of melancholic MDD. Abnormal ReHo in the right middle temporal gyrus may play an important role in the pathophysiology of non-melancholic MDD. In addition, a combination of the ReHo values in the right fusiform gyrus/cerebellum Crus I and the right superior occipital gyrus/middle occipital gyrus might be employed as a potential imaging marker for separating patients with melancholic MDD from patients with non-melancholic MDD. And a combination of abnormal ReHo values in the right fusiform gyrus/cerebellum VI and left postcentral gyrus/precentral gyrus might be a potential imaging marker for separating melancholic MDD patients from healthy controls.

## Data Availability Statement

The raw data supporting the conclusions of this article will be made available by the authors, without undue reservation.

## Ethics Statement

The studies involving human participants were reviewed and approved by the Medical Research Ethics Committee of the Second Xiangya Hospital of Central South University, China. The patients/participants provided their written informed consent to participate in this study.

## Author Contributions

All authors listed have made a substantial, direct and intellectual contribution to the work, and approved it for publication.

## Conflict of Interest

The authors declare that the research was conducted in the absence of any commercial or financial relationships that could be construed as a potential conflict of interest.

## Supplementary Material

Supplementary Figure 1 The ventricular seed-based region of interest (ROI) in data preprocessing.

Supplementary Table 1 Significant ReHo differences across three groups^*^.
