# Supplementary figures and images for: Disrupted Regional Homogeneity in Melancholic and Non-melancholic Major Depressive Disorder at Rest
Source: Front Psychiatry. 2021 Feb 16;12:618805. doi: 10.3389/fpsyt.2021.618805 (PMC7928375; doi:10.3389/fpsyt.2021.618805)

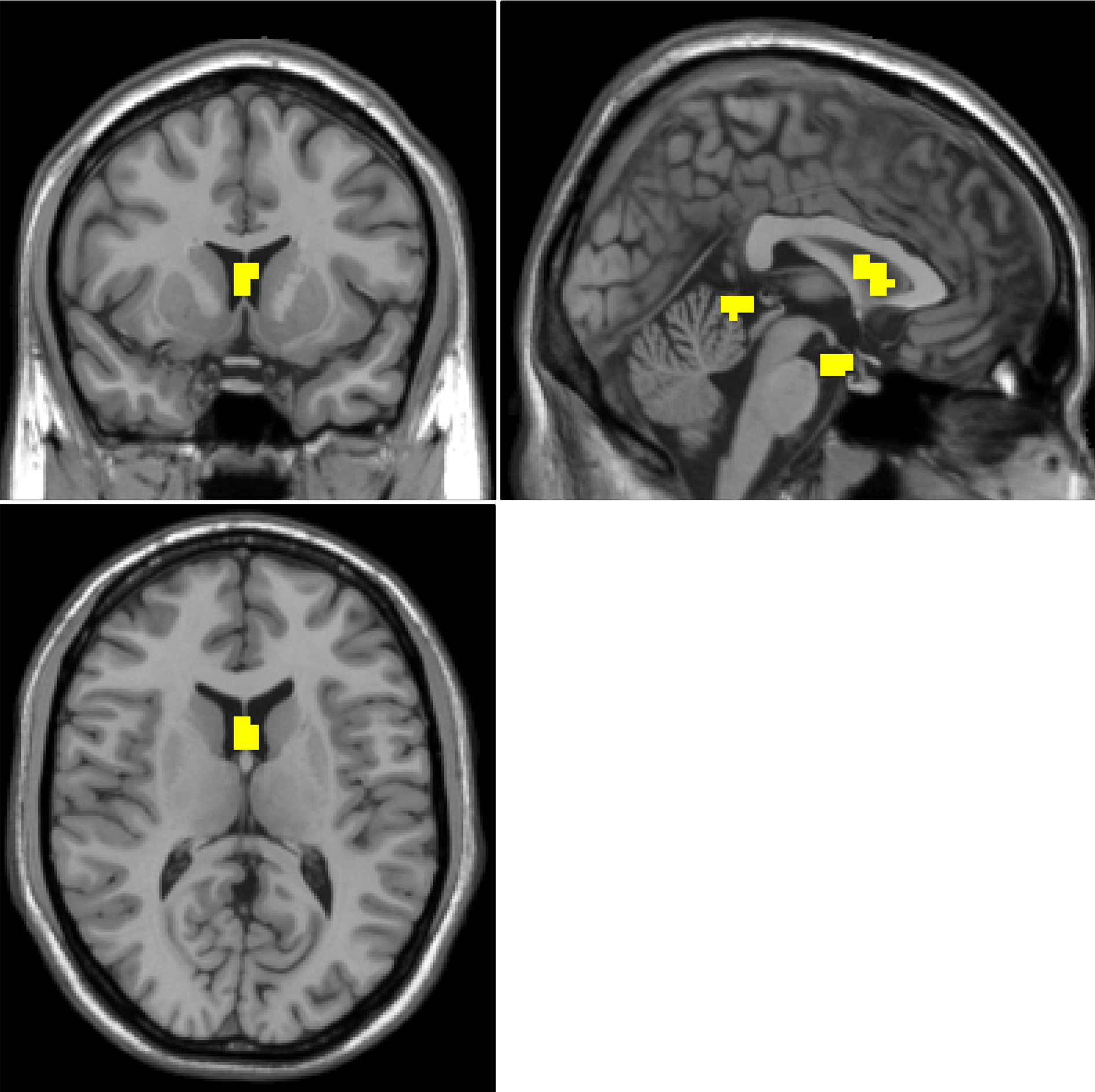

Supplement: Supplementary Figure 1 — The ventricular seed-based region of interest (ROI) in data preprocessing. [file Image_1.TIF]
