# Supplementary material for: Disrupted Regional Homogeneity in Melancholic and Non-melancholic Major Depressive Disorder at Rest
Source: Front Psychiatry. 2021 Feb 16;12:618805. doi: 10.3389/fpsyt.2021.618805 (PMC7928375; doi:10.3389/fpsyt.2021.618805)
Supplement: Supplementary Table 1 — Significant ReHo differences across three groups*. [file Table_1.DOC]

Table S1. Significant ReHo differences across three groups*.

| Cluster location | Peak (MNI) | | | Number of voxels | *T* value |
| --- | --- | --- | --- | --- | --- |
|  | x | y | z |  |  |
| *Melancholic vs Non-melancholic* |  |  |  |  |  |
| Right Middle Frontal Gyrus | 42 | 39 | 36 | 39 | 3.3322 |
| Right Fusiform Gyrus/Cerebellum Crus I | 42 | -48 | -24 | 48 | -4.2235 |
| Right Superior Occipital Gyrus/Middle Occipital Gyrus | 24 | -90 | 33 | 76 | -4.1056 |
|  |  |  |  |  |  |
| *Melancholic vs Healthy Controls* |  |  |  |  |  |
| Bilateral Cerebellum Crus II | -9 | -90 | -33 | 49 | 4.0705 |
| Right Fusiform Gyrus/Cerebellum VI | 30 | -63 | -15 | 65 | -4.5494 |
| Left Middle Occipital Gyrus/Inferior Occipital Gyrus | -36 | -75 | 3 | 90 | -4.1533 |
| Bilateral Superior Occipital Gyrus/Middle Occipital Gyrus | 18 | -87 | 21 | 336 | -4.5307 |
| Right Postcentral Gyrus/Precentral Gyrus | 63 | -9 | 15 | 30 | -3.5494 |
| Left Postcentral Gyrus/Precentral Gyrus | -54 | -9 | 21 | 63 | -4.5727 |
|  |  |  |  |  |  |
| *Non-melancholic vs Healthy Controls* |  |  |  |  |  |
| Right Middle Temporal Gyrus | 66 | -51 | 3 | 36 | 3.1798 |

* Age, years of education, gender, and framewise displacement were used as covariates

MNI = Montreal Neurological Institute.

ReHo=regional homogeneity
